# Dental Remedies

**Published:** 1908-04-15

**Authors:** J. P. Buckley

**Affiliations:** Chicago


### DENTAL REMEDIES.

BY DR. J. P. BUCKLEY, PH.G., D.D.S., OF CHICAGO.

An Address delivered before the First District Dental Society, of Detroit, Michigan.

Dr. Spalding in his remarks has incidentally referred to the fact that this is the third year that this Society has had a Chicago man as its guest. I have always been proud of the fact that I was a dentist and there was a time in my life when I harbored a feeling that it was a privilege to practice my profession in the City of Chicago; but a few years ago I was the guest of the South Dakota State Dental Society at a time when our city was enjoying one of its characteristic labor strikes, and the city was being advertised throughout the country in a manner not altogether to her credit and while I was in this little Dakota city, the President of their State Society introduced me to a distinguished appearing gentleman, as Dr. Buckley of Chicago. The gentleman grasped my hand, in a most cordial and friendly fashion, and after expressing his pleasure at meeting me, said; "Well, which are you, an anarchist or a socialist?" This was quite enough to arouse my animosity, and as I was about to reply to him rather sarcastically, but looking him in the eye and discerning an honest and sincere look, I smiled and said that I was neither, that the population of the city of Chicago was divided largely into three great classes—Anarchists, Socialists and Dentists, and that I had the honor to belong to the latter class.

I have had occasion to speak before a great many dental societies in this country; but I remember of none where the Society was graced by the presence of ladies, as is this one. You know the women do take an advanced stand today; they are rightfully making their way to the front. Now I do not live in Chicago; I live just outside of the limits, in a suburb known as Oak Park, and, while all women are aggressive, we feel that we have the most aggressive women in our little suburb of any part of the United States. To illustrate how aggressive they are, I will relate a little incident that happened the other day. A book agent came to the door and little Johnnie, six or seven years old, answered the door bell. The agent said:

"How do do, sonny. Is the head of the house in?"

Johnnie said: "No sor, nobody's home but me fadder."

If the women of Oak Park would grace our suburb by doing the work that I saw done by the ladies of this Society in your Clinic today, there would be one man in that suburb that would not object to the progress that they are making.

If there is anything to-night that I want to emphasize, it is the importance of preventing pyorrhea; and there is a way to prevent it, and that way was well demonstrated at your Clinic to-day. It seems almost misfitting to interrupt this jovial meeting by talking "shop," but that is what you have asked me to do. I would rather go on telling more stories, but the hour is late and I must say something pertaining to my subject.

The subject assigned to me is "Dental Therapeutics." Before I talk on Dental Therapeutics or the application of drugs to the treatment of disease, I want you to have in your minds the definition of two or three terms that I have in mind. You perhaps may be familiar with these terms, as those of modern thinkers, without any suggestion on my part, but I will simply review them so that if I mention a word you will know what I mean.

I prefer to think of Materia Medica as including everything that we know about drugs from their physical and chemical aspects, therefore Materia Medica is a kind of history of drugs. The other two things that I generally expect to be studied besides their general history are, the action, and the application of the drug. We have men to-day who are spending their entire time investigating the action of drugs and remedies upon the normal tissues and organic functions of the body. In other words, we are developing a science known as pharmacology. Pharmacology should not be confused with the science of drugs; called Materia Medica. Pharmacology includes only the action of drugs upon the normal tissues of the body; the science of the application of drugs and medicines to the treatment of disease is known as Therapeutics. Successful Therapeutics must necessarily rest upon Materia Medica and Pharmacology.

In order to be able to successfully apply drugs and medicines to the treatment of disease, we must be able to recognize pathologic condition at once. This means that we should have a knowledge of general pathology, as well as an accurate knowledge of dental or special pathology. The first essential, then, is to become familiar with the nature of the more prevalent diseases, so as to be able to differentiate pathological conditions. After a diseased condition is recognized we must know, if we are going to be successful in our therapeutics, what drug or remedy, is indicated and which if applied in a certain way or form, or by a certain method, will react upon that abnormal condition most favorably. In fact we do not expect a drug or remedy to cure any disease, but we ought to know what drug or remedy will best aid in overcoming that abnormal condition. An essential of all successful therapeutics consists in the finding of a practical form of drug or remedy which we know, or believe from past experience and observation, will reach that abnormal condition and act upon it most favorably. This means, then, that we have got to have some knowledge of pharmacy as well as pathology and Materia Medica. We should be familiar with certain physical and chemical formulas and their application in pharmacy and therapeutics. In the time at my disposal I shall be unable to say much about pathology, and what I say will be but incidentally. I want to call attention however to a few practical drugs and remedies that it seems to me dentists ought to know more about, and I will try to emphasize or illustrate a few practical laws in pharmacy and chemistry which must be understood and applied in a practical way to succeed.

The first thing to which I shall call your attention is oral hygiene, and I will be criticized perhaps for doing so because of my rudimentary treatment. In Chicago we take off our hats just as gracefully as we can to some of you men in Michigan who are blazing the way in oral prophylaxis. To my mind that is the most important subject before the dental profession to-day,—and I do not except the cast gold inlay process. I have one adjunct to oral prophylaxis which may seem rudimentary, but it is the one remedy used in my office with perhaps the most satisfaction of anything that I use. The remedy I value so much is only an essential oil water. I should not take the time to make this water before you and impose upon your good nature if it was not for the fact that so few dentists know how to make it. A teacher in one of the prominent colleges in Chicago, at a public Clinic, was showing how to make an essential oil water, he said: "Take a few drops of the oil and drop it in a bottle of water and shake it well." Now you cannot make a good remedy that way that I would recommend for your use in prophylaxis work. To make an essential oil water you have to take some insoluble powder, it does not make much difference what you use so long as it is a clean insoluble substance. All the essential oils are insoluble in water, and to make the official essential oil water your object is to get the water to take up as much of the oil as possible, and the best way to do that is to divide the globules of the oil so the water can get at them. We do this by taking, according to the pharmacopoea, one drachm of phosphate of calcium or magnesium carbonate, I have precipitated calcium phosphate here, and to one drachm we will add 15 minims of the essential oil. I have cinnamon oil here, which I use most because my patients like it. I use this for three or four days, in the spraying apparatus, or have it in a glass on the bracket to rinse out the pyorrhea pocket as I am scaling the teeth. Then I may want a change, and I have my assistant make a peppermint water, or once in a while have her make a wintergreen water. If the peppermint water is made I usually add a half grain of saccharin to the half pint of peppermint water. Add 15 drops of Oil of Cinnamon to 30 grains of Precipitated Calcium Phosphate and triturate in a mortar until thoroughly mixed. Then add eight ounces of distilled water and filter. I don't want you to go down to the drug store and buy this. You can buy it and it is cheap, but I want you to have your assistant make it and I want you to use it just as though it was only water, and stop buying fake antiseptic solutions. If it is filtered without previously wetting the filter paper, the solution will run through milky, but if it is simply wetted, then it will run through clear. Now this is the best of all the mouthwashes that is used in my office; it is also the vehicle for all local anaesthetic solutions used. In scaling pyorrhoea you cannot do better than to have your instruments in such an antiseptic solution; there is no need of having them in a disinfectant solution. There might be some need of it in some cases but you cannot always do it; you have to be practical in therapeutics as well as practical in your operative and prosthetic dentistry. We cannot use the strong disinfectants in the mouth. All that we can use for mouthwashes, or to keep our instruments in after they are once sterilized, is an antiseptic solution. This will be an antiseptic solution; it is pleasant, and patients like it. I often have patients remark that "no other dentist ever did that to me; what is that—it tastes good." Keep your instruments in this solution when scaling the teeth, it will be better for this purpose if you add a drop or two of "modified phenol," that is phenol cut with a little alcohol. One of the best mouthwashes that you can give your patients is this solution. If you need an astringent mouthwash, any astringent that is soluble in water may be added to it. Dr. Whitsler of Cleveland, has recently called our attention to zinc phenol sulphonate, which is an excellent astringent which can be dissolved in cinnamon water.

I know that the pulp can be removed by anaesthetizing it, without the tooth getting sore, but if you use the devitalization method I advise that you use the prescription that I have on the board (No. 1. See below) which you will find an excellent one. You ought to know something about the compounding of that prescription, otherwise you will not be satisfied. You can suggest to your pharmacist, without any impropriety, that when he mixes that prescription he use just as small an amount of lanoline as possible and still make a stiff paste with the other ingredients. The basis of this prescription is arsenic trioxide; which is the same preparation formerly known as Areneous Acid. Cocaine Alkoloid is insoluble, but as our vehicle for this paste is a fat or oil it will dissolve the cocaine, so we can use alkaloidal cocaine instead of the alkaloid salt. The menthol is added largely for a pharmacal reason—the menthol will take up the water in the lanoline and liquefy the five grains of the menthol, with a small amount of lanoline it will be sufficient to make a nice stiff paste out of the arsenic and the cocaine. I call your attention to this, because the druggist, in filling this prescription will use the ordinary amount of lanoline and your paste will be too slow in its action and you will not like it. Place this devitalizing paste on the pulp and fill the cavity as usual and leave it in as long as from your experience you should leave it to accomplish the purpose, then you take out the filling and open freely into the pulp chamber, do not attempt to remove all the pulp at this time but seal in some remedy,—and the remedy that I like to use, and I will suggest that you try, is the anodyne remedy (No. 2. See below), because it contains thymol, seal this in until the next sitting, when you can remove all the pulp aseptically and fill the roots. I believe that this is the best method of removing pulps by the devitalization method.

I would call your attention to a change in the new pharmacopoea which has changed cocaine hydrochlorate to cocaine hydrochloride. When buying cocaine get the flake cocaine hydrochloride, I like it because it melts down like freshly fallen snow. Saturate a pledget of cotton with the antiseptic solution and then touch to this flake cocaine hydrochloride and it will quickly dissolve making a convenient means of applying. cocaine when a definite solution is not required. You cannot always get the best results by such a solution; especially if you have to force the solution through the dentine when you do not have an exposure, by making the solution only four or five per cent it can be more readily forced through the tubuli.

When you have removed the pulp, unless you follow the method of removing the pulp by devitalization to which I have called your attention, an anodyne treatment is essential. Especially is this true when you have removed your pulp by pressure anaesthesia. The object has been to force the anaesthetizing solution only to the pulp end. This is easier said than done, however. The object has also been to remove the pulp aseptically, which is more easily accomplished. No matter how careful you were in removing the several fragments, when you removed that pulp after it was anaesthetized you have simply torn live tissue from live tissue. I know in some instances it is exceedingly small; in other instances it is larger than we hoped that it might be, but you have simply torn live tissue from live tissue, and if you have not irritated the tissue beyond the end of the root by forcing the anaesthetic solution too far, you have never-the-less more or less mechanically irritated the apical tissue, and the thing that is indicated in that root canal is an anodyne, I know of nothing that is better than this remedy. It is like some other anodynes, however, it can be used too much and will have a tendency to do more than to allay; it will then slightly irritate,—and, knowing that in advance, we do not need to use too much of the remedy.

If the canal is not dry and you seal into it dry cotton, the cotton will absorb the moisture from the apical end, and just so when you place this anodyne remedy in the chamber a little dry cotton should first be packed into the apical third of the root to prevent the remedy from passing through in excess to irritate the inflamed tissues. There are three things to be observed in removing live pulps and the subsequent treatment: First we should establish and maintain asepsis in the entire operation; secondly we should preserve the color of the tooth by the drugs we use. If the tooth is discolored when the pulp is removed, it is our fault, and is not due to the fact that the pulp has been removed. The third thing is to prevent subsequent infection by a proper root filling.

I use the Goodrich white, instead of pink gutta percha for filling roots. Use anything you have success with so long as it is not a proprietary remedy. I like to use gutta percha, because I have learned to use it and because I have good success in using it. I cut it with chloroform and make chloropercha, and then add to it the modified eucalyptol (See formula No. 3) and place it in a bottle leaving the stopper out and letting the chloroform evaporate, which leaves the gutta percha in the bottom of the bottle in a lump. If you have gutta percha in this form and have an ordinary root to fill, take a little piece out of the bottle and put it in the canal, and if it don't melt down soon enough, take a burnisher and put it in the flame and simply touch it to the gutta percha and if the canal is dry, as it ought to be, it will run right in the canal, and you can then force to place the gutta percha root cone which approximately fills the root. Sometimes we get careless in removing live pulps from the teeth or in filling the roots, and occasionally the tooth will get sore; that is, they do once in a while in my practice. I admit that, and I presume that occasionally they get sore after you fill them. Something different takes place, it may be the susceptibility of the patient. Your stand-by in such an event has been tincture of aconite and tincture of iodine, or some such remedy, as iodine and chloroform. If you use aconite you should know that the pharmacopoea has put all tinctures on the 10 per cent basis; tincture of aconite was formerly 35 per cent in strength, in the last pharmacopoea published in 1905 the strength was reduced to 10 per cent, so if your original formula for that celebrated counter-irritant was equal parts of aconite, iodine and chloroform you should treble the amount of the aconite at least, and then you will not have the same remedy that you used to have, because you will have more alkali in your solution even though you treble the amount of aconite. It is not always that we care to have a counter-irritant action. The counter-irritant does three things; it irritates the normal part superficially for the purpose of influencing favorably the diseased part, which is usually deep-seated; and draws the blood away from the diseased part by diversion and by acting on the vasomotor nerves; if there is infection it wakes up the sluggish leucocytes in that particular locality to carry off the infectious material; it produces a psychic effect upon the mental side of the patient, which stimulates the vital forces. In some cases a liniment will act better than a counter-irritant as it produces longer stimulation, if that is what you need you can use the No. 4 formula below.

There are to-day too many empirical uses of drugs in dentistry. Empiricism in dentistry has been prominent in the past, but if there is any one place more than another where dentists seem to ignore the rationalism of the method they are using, or trying to use—for some dentists find out as little about a result so long as they can and be safe—it has seemed to me to be in the treatment that putrescent pulps have received. There is a tendency in dentistry, I am glad to know, as well as in medicine, to learn the why and wherefore, and that is one of the reasons that we are being recognized a true and distinct profession. Some dentists like to be classed as specialists in medicine. I would just as soon be classed as a "Dentist" or a Doctor of Dental Surgery, because I prefer to belong to a true and distinct profession. We are now being recognized by the medical profession more than ever before, and also by the general public for what we know and do. We shall be recognized with more and more of credit if we inject more rationalism into our methods in handling drugs and chemicals, for the therapeutic practice of dentistry is the nearest that we come to being medical specialists. When you attempt to treat a putrescent root canal you should do it with your eyes open to all the conditions involved, otherwise you are likely to be working in the dark, you ought to know the nature of the contents of that root canal. How can you go to your drug case and select a drug or remedy with any intelligence to change conditions the nature of which you know nothing? The first thing that I would suggest is a careful study of the possible features that may be prevalent that the pulp undergoes during the process of decomposition. We must know that something is there in the form of gases, because when you hermetically seal the root your tooth will ache from the pressure generated, or we may surmise that something we do not know what, has been forced through the end of the root, and an alveolar abscess is threatened. We have all experienced this difficulty. But we think we know something of the nature of pulp decomposition from infection. We believe we can recognize certain conditions. There are undoubtedly ptomaines formed that are highly poisonous, at least some of them are; there may be some carbon monoxide resulting from the decomposition of the carbo-hydrate constituents; ammonia or its derivatives, resulting from the decomposition of the proteid material or the further decomposition of the ptomaines; probably there is some hydrogen sulphide or a derivative from that particular gas. Now we have one remedy which has proven to be especially successful in the treatment of putrescent pulps. That agent is Formaldehyde. There probably is no doubt but that most of these gases that are generated ultimately result from the decomposition of the pulp, and you should seal into the tooth something that will unite with them and form some other compound. There is constantly generated in the decay of the pulp two gases, ammonia and hydrogen sulphide. Formaldehyde is a gas which will unite with hydrogen sulphide and form wood alcohol and sulphur, it will also unite with ammonia gas and form urotropin and water. If these two gases, ammonia and hydrogen sulphide have had anything to do with your troubles in the past especially with the pressure that was created when hermetically sealed the canal which con- tained the decomposing pulp then logically formaldehyde gas is especially indicated. When the venders of proprietary preparations say they can hermetically seal their preparations in the canal, they must have something in them from which formaldehyde gas or a similar reagent is generated. These gases must be changed into inert liquids and solids, and if you convert the gases chemically into inert liquids or solids, of course you can hermetically seal the cavity without danger of having pressure generated from the formation of gases which would force through the root and produce pericemental trouble.

I do not know of anything that is more irritating than formaldehyde gas and it must therefore be carefully applied, but if there are putrescent gases in the root canal this effect will be nutrilized. You can modify the intense action of the formaldehyde by using other drugs with it. You can combine beech wood creosote and formalin in equal parts with a few drops of alcohol added to make a clear solution. Ten minims of alcohol is sufficient to each drachm that you take of the creosote and formalin, this makes a nice clear solution. If you do not want to use creosote, you can take equal parts of phenol and formalin and they will mix better than the creosote, formalin and alcohol. Tricresol is a refined mixture and colorless of three cresols; meta, ortho and para cresol. It was formerly called tricresol, but is now recognized in the new pharmacopoea as cresol, but if you go to the druggists they will try and give you liquor of cresolis. Liquor of cresolis is a substitute for lysol and is reddish brown in color. It contains potassium hydrate, linseed oil, cresol and something else that I have forgotten. It is not the drug you want at all, insist on having what is now known as cresol and was formerly known as tricresol. When this remedy is mixed keep it in an amber colored bottle. Tricresol or cresol will mix with formalin in all proportions. You may not think so at first, but when you shake it the solution will become clear. One reason why I like cresol better than creosote, is that it will mix with formalin in all proportions, thus making a good pharmacal preparation. Phenol is better than creosote, but cresol is a better germicide than phenol. All three of these drugs have more or less analgesic properties, and it is this property that counteracts the irritant tendency of the formaldehyde, because it is constantly being generated from the formula. Forcing this remedy through the end of the root will make the patient more uncomfortable than anything I know of, but that would be foolishness, not to say carelessness on the part of the dentist. When you have a putrescent pulp open into the chamber freely, and on a small pledget of cotton dipped in the remedy place it in the pulp chamber and hermetically seal it with cement. A few years ago if some of you had told me to do that I would have thought you were crazy.

You have got to do two things, in using this remedy to get satisfactory results. When you open into the pulp chamber and put this remedy into that pulp chamber you must hermetically seal it with cement. I trust it is obvious that you ought to open into the pulp chamber freely, so as to give this remedy, which constantly generates formaldehyde gas, a chance to get into all the canals where putrefaction is taking place, so that it will act on all the gases generated there, and neutralize them in all the canals, not in one or two, for instance, and leave one, but convert all the gases into liquids and solids so that you can hermetically seal the tooth without its aching. You can do this if you freely open into the pulp chamber. The other thing you have to do, besides opening up all the chambers and giving the remedy a chance to act on the contents of all, is to hermetically seal it in the tooth *without pressure*. I know that most of you, if you are careful, can do that with the temporary gutta percha, but you cannot prevent your patients from going home, and because the tooth is sore five or six hours afterwards, chewing on that side of the mouth the food thus forcing that filling down into the cavity, and forcing the remedy through the putrescent material and on through the canal, before the chemical reactions have taken place, setting up a violent tooth ache. It is not necessary to fill the entire cavity with cement, you should have a veneer of cement. You can fill the bulk of the cavity with cotton if you like, and then take any cheap hydraulic cement and make a veneer over that cotton, and then, when your patient comes back, you can drill a little hole into the veneer cement on the occlusal and remove that cement filling just as easy as removing any ordinary filling. To be on the safe side, it is best to use this remedy with cement.

If the tricresol turns red it is more liable to discolor the tooth.

I want to commend a remedy to you to be used in the treatment of complicated alveolar abscesses. If you have a condition where putrescent material has forced itself through the end of the root and the tissue in the apical area has been broken down and you have the denuded end of the root with which to contend, or have a root which is abraded and with rough edges, or if pus has burrowed its way through the alveolus and the edges of the fistula is rough, if you expect to cure that kind of an abscess by sealing this or any other remedy within the tooth structure only, you are expecting too much of drugs and medicines, and that is the one thing that many dentists too often do. Some of us expect one remedy, if it is good in one place, to be good everywhere and we use it for every condition. We expect too much of drugs. The only thing that you ought to expect of a drug or remedy is to have it aid nature in her effort to overcome some abnormal condition, nature does the cure. Where you have the condition to which I alluded, an absorbed root end, or denuded end of the root, or where you have a sluggish condition in the apical area, you have got to either treat it surgically or burn it out with irritant caustics, or else stimulate the tissue and cause the sluggish or chronic condition to become more acute. I think just as much of phenol sulfonic acid for this purpose as I do of this cresol and formalin for putrescent pulps. Phenol sulfonic acid is made of phenol and sulphuric acid in equal parts to which you add as much water as you use of both the phenol and sulphuric acid. You cannot make this remedy by simply combining mechanically the drugs, there is a little pharmacy to consider as phenol sulphonic acid will not result from simply putting these two drugs together. Take phenol 95 per cent, one drachm and chemically pure sulphuric acid,—some have been recommending crude phenol and crude sulphuric acid. If you mix those two you have a black solution that ought to be thrown in the cuspidor as soon as it is mixed. Heat the phenol in the test tube or evaporating dish or beaker nearly to boiling, and then while hot gradually add the sulphuric acid, and while this solution is still hot gradually add as much water as you have taken of both phenol and sulphuric acid. If you use lactic acid for treating pyorrhea you will find phenol sulfonic acid a better remedy. If you have a bad pyorrhoea pocket, one where you have scaled the root properly, or there is not much to scale because of an abscess, and the pericemental membrane is rapidly being destroyed, and you seem to have difficulty in stopping the flow of pus, a 25 per-cent solution of phenol sulphonic acid syringed into the pocket slowly as you remove the syringe from the pocket, will literally cook the entire area of the pocket, and if you have done your surgical duty first you will not see much pus there from that time on. It is not severely caustic, as you might expect. It is what you would classify as a stimulating caustic. In those cases where you have a chronic alveolar abscess with an established sinus, through which your practice is to force phenol or modified phenol, or some anodyne remedy, neither of them will have any action except to lessen the pain; but if you use phenol it will simply cauterize that with which it comes in contact. If you need to force any such drugs through wounds once, or twice or three times, do not use these remedies, but specify phenol sulphonic acid. After you use a fifty per cent solution in a real difficult case you could use the burr. If you wish to put this remedy in the pulp canal use it on threads of silk or asbestos. If you use cotton, especially with the pure remedy, and often with the fifty percent solution, the acid will simply make a mush out of the cotton and then you will force the remedy through the apical opening cotton and all.

I do not know how many mouthwashes there are in the market. There are probably as many mouthwashes as there are abscess killers and there may be as many as there are hypnotic headache remedies. The pharmacopoea among the proprietary headache remedies introduced a formula or remedy the composition of which we know; it combined acetanilid powder—acetanilid, caffein and sodium bicarbonate. They also, in order to offset the rapid influx in medicine of antiseptic solutions, the composition of which we did not know, introduced an official antiseptic solution, called *Liquor Antisepticus*, which is similar in character and action as Listerine.

Boric acid,
Benzoic acid,
Thymol,
Eucalyptol,
Oil of peppermint,
Oil of Wintergreen,
Oil of Thyme,
Alcohol, and water.

Knowing this is recognized by the pharmacopoea you can write a prescription for it by its official title Liquor Anti septicus.

When you have scaled the teeth in your prophylactic work you can spray with the cinnamon or peppermint water, and your patients will like that. If you want a stronger antiseptic you can spray the mouth out with this antiseptic solution. You can not prescribe a strong disinfectant for your patients with safety, but you can prescribe an astringent, antiseptic mouthwash made from this solution. The prescription which I nearly always write now, when the patient's teeth have been scaled in pyorrhea, is this: zinc sulpho-carbolate, zinc phenol sulphuate, dr. ½ each and liquor antisepticus fluid ounces 8. Patients to use this solution in water at home. It seems to me that it is more intelligent to write a prescription for this, or some other good mouthwash the contents of which you know and it displays more intelligence than to simply sign your name to a printed blank which is furnished you by the various proprietary manufacturers. A parrot could do that, or even your office girl. The proprietary men by sending those so-called blank prescriptions to your office insult your intelligence. If you prescribe liquor antisepticus it shows that you know it is a recognized solution and it is good.

I submit a few good dental formulae which I shall be unable to discuss for you tonight, but which you can use with good results if you will take the trouble to copy them and take them home with you for future study and use.

DEVITALIZATION PASTE. (No 1.)

R Arsenic Trioxide................ ʒ j
Cocaine........................ gr. xx
Menthol........................ gr. v
Lanolin, add q. s. to make paste.

Note—Lamp-black can be added to color paste if desired.

ANODYNE REMEDY. (No. 2.)

R Menthol........................ ʒ j
Thymol......................... ʒ ij
Phenol......................... ʒ iij

MODIFIED EUCALYPTOL. (No. 3.)

R Thymol................... grs. ij
Menthol.................. grs. iij
Eucalyptol............... ʒ j

LINIMENT. (No. 4.)

R Menthol.................. grs. xx

Chloroform............... ʒ ij

Tincture Aconite, 10%...... ʒ vj

LINIMENT. (No. 5.)

R Tincture Aconite ʒ ij
Tincture Iodine
Chloroform aa ʒ j
Sig. Dry membrane and apply freely over affected tooth.

NEURALGIA.
(From *Dental Cosmos* )

R Quininae valerianatis gr. xxiij
Extracti hyoscyami gr. iv
Extracti cinchonae gr. viij
M. Fiat Piulae No. 12.
Sig. Take one pill at meals and on retiring.

PUTRESCENT PULPS. (No. 6.)

R Formalin,
Creosote aa ʒ j
Alcohol M XX
Mix, and use as directed.

PUTRESCENT PULPS. (No. 7.)

R Cresol

Formalin aa ʒ j

Sig. Use as directed.

(No. 8.)
(*Talbott's Formula.*)

R Iodide of Zinc gr. xv
Water mx
Iodine Crystal gr. xxv
Glycerine ʒ j
Sig. Use as directed.

LOCAL ANAESTHETIC. (No. 9.)

R Cocaine hydarochloride gr. vj
Phenol mij
Aquae Menthol ad ℥ j
Use as directed.
(19 minims of this solution contains ¼ grain of the alkaloidal salt.

ASTRINGENT SOLUTION. (No. 10.)

R Zinc phenolsulfonate ʒ ss
Liquor antisepticus ℥ viij
Sig. Astringent mouth-wash spray.

NEURALGIA.

R Pulvis acetanilidi comp gr. xij
Fiant chartulae No. 2.
Sig. Take one powder at once and the other in two hours if necessary.

U. S. P. FORMULA FOR ACETANILID COMPOUND POWDER.

Acetanilid ........ 70 parts
Caffein ........ 10 parts
Sodium Bicarbonate ........ 20 parts

NEURALGIA.

R Salophen,
Phenacetin ........ aa gr xx
M. chartulae No. 4.
Sig. Take one powder every two hours as required.

MODIFIED PHENOL.

R Menthol ........ ʒ j
Thymol ........ ʒ ij
Phenol ........ ʒ iij

ANODYNE PASTE.

R Orthoform ........
Europhene ........ aa ʒ j
Liquid Petroleum to make thin paste.
Sig. Apply to abrasions or painful tooth pockets.

